# Transmitter and Receiver Circuits for a High-Speed Polymer Fiber-Based PAM-4 Communication Link

**DOI:** 10.3390/s22176645

**Published:** 2022-09-02

**Authors:** Frida Strömbeck, Mingquan Bao, Zhongxia Simon He, Herbert Zirath

**Affiliations:** Microwave Electronics Laboratory, Chalmers University of Technology, 41258 Gothenburg, Sweden

**Keywords:** DHBT, energy efficient, InP, modulator, PAM, PD, PMF, polymer microwave fiber, power detector, pulse amplitude modulation, RF-DAC

## Abstract

A high data rate RF-DAC and a power detector (PD) are designed and fabricated in a 250 nm indium phosphide (InP) double heterojunction bipolar transistor (DHBT) technology. A communication link using the Tx-Rx over polymer microwave fiber (PMF) is measured. The link consists of a pulse amplitude modulation (PAM) modulator and a PD as a demodulator, as well as a one-meter-long dielectric waveguide. The working frequency range of the complete link is verified to be 110–150 GHz. The peak output power of the PAM modulator is 5 dBm, and it has a −3 dB bandwidth of 43 GHz. The PD consists of a parallel connected common emitter configured transistor and a common base configured transistor to suppress the odd-order harmonics at the PD’s output, as well as a stacked transistor to amplify the output signal. Tx and Rx chips, including pads, occupy a total area of only 0.83 mm^2^. The PMF link can support a PAM-4 signal with 22 Gbps data transmission, and a PAM-2 signal with 30 Gbps data transmission, with a bit error rate (BER) of <10−12, with demodulation performed in real time. Furthermore, the energy efficiency for the link (Tx + Rx) is 4.1 pJ/bit, using digital data input and receiving PAM-2 output (5.6 pJ/bit for PAM-4).

## 1. Introduction

On the emergence of the internet of things (IoT), autonomous driving, and virtual reality applications, the number of interconnected devices and the throughput of connections between such devices are increasing exponentially. This is especially true for real-time imaging and video capture devices, for which several to tens of Gbps throughput is required. For example, current cars with autonomous driving features are already equipped with multiple high-definition cameras, and the video has to be aggregated and processed centrally [1]. Currently, fiber optic solution is the main high data rate connectivity solution for cars; however, high data rate opto-electronics are expensive and sensitive to high temperature. Another use example lies in the telecommunication field, where distributed antennas in the 5G base stations require cost-effective connectivity solutions to processor units with above 40 Gbps data rates.

Millimeter-wave modulated signals transferring over polymer microwave fibers (PMF) is a promising solution for high-speed connectivity. The PMFs are flexible and made of low-cost plastics. Compared to optical fibers, the alignment to the PMF is much simpler, because of the larger size of the PMF. Furthermore, Millimeter-wave semiconductor processes such as SiGe BiCMOS and InP DHBT can be certified for automotive or even space temperature grade, which makes the PMF a more robust solution in comparison with fiber optic. The modulated signals are mostly confined in the PMF; therefore, there are no legal frequency restrictions, which either limit the data rate or require high modulation schemes, thus requiring high complexity, and therefore high cost and high power consumption.

Several PMF links have already been demonstrated: A QPSK transmission over 4 m with a data rate of 4 Gbps was presented at 60 GHz [2]. Using continuous-phase frequency-shift keying (CP-FSK) at 120 GHz center frequency, a high data rate of 17.7 Gbps was demonstrated over one meter [3]. In this work, a PAM-4 modulated PMF link is demonstrated. It is measured to support data rates up to 30 Gbps over one-meter PMF. It covers large parts of D-band (110–170 GHz) and requires no carrier recovery, lowering the complexity of the transmitter and receiver.

Demodulation of an amplitude-modulated signal is performed with a power detector (PD). The design of the PD in this work was based on insights gained from previous work [4], which can support demodulation of an amplitude-modulated signal up to 14 Gbps. The design topology contained two passive baluns, which are made of coupled transmission lines. A side effect, noticed in the previous work, of using coupled transmission lines together with existing resistance in the circuit is that it can increase response time and result in distortion of the shape of the output waveform (for example overshoot). In this work, the use of a passive balun is eliminated to avoid these issues and improve the speed of the PD.

**Figure 1:**
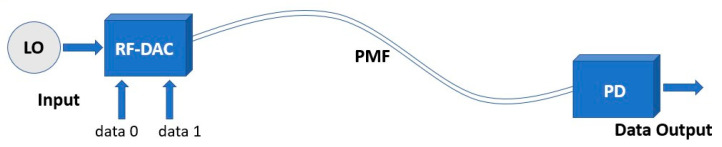
Overview of the PMF link.

The PMF link consists of three different parts: a radio frequency digital-to-analog converter (RF-DAC), a rectangular high-density (with a density above 0.930 g/cm^3^) polyethylene (HD-PE) fiber acting as a flexible dielectric waveguide, and a power detector (PD), which receives the signal and demodulates it. A simplified illustration of the PMF link can be seen in Figure 1. An LO at desired output frequency is applied to the RF-DAC, where the data modulate the signal into an amplitude modulated signal. The signal is then transmitted over the PMF to a power detector (PD), where the signal is demodulated.

To realize the transmitter and receiver, high data rate monolithic microwave integrated circuits (MMICs) are designed and fabricated using indium phosphide (InP) double heterojunction bipolar transistor (DHBT) technology with 250 nm emitter width developed by Teledyne Scientific Company. In this process, transistors can operate at an emitter current density of up to 12 mA/µm^2^ with a maximum collector–emitter voltage of 2 V. Measurements on a transistor with 10 µm emitter length indicated an extracted *f*_t_ and *f*_max_ of 350 and 600 GHz, as well as a minimum noise figure of 4.3 dB [5].

The structure of this paper is divided as follows: after the introduction in Section 1, information about the design of the RF-DAC-based PAM-4 modulator is given in Section 2. Section 3 covers the design of the power detector. The combined measurement results are presented in Section 4. A discussion and conclusion are given in Section 5.

## 2. Design of the RF-DAC-Based PAM-4 Modulator

**Figure 2:**
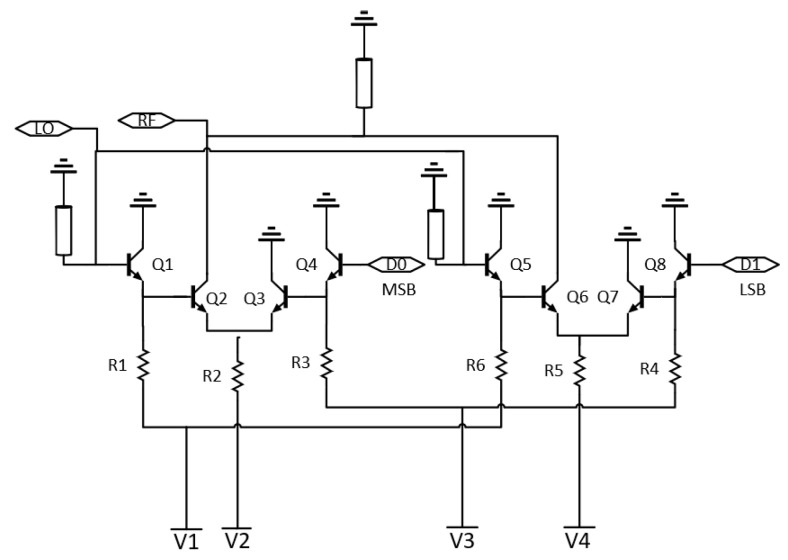
A simplified schematic of the proposed RF-DAC-based PAM-4 modulator.

The topology uses emitter-coupled pairs (ECP), which function as a switch, to enable low power operation as well as fast switching (high data rate). An LO input is modulated to a PAM signal through different levels of amplification/attenuation applied to the signal. Furthermore, two ECPs are used for the PAM-4 signal. One pair is used for the most significant bit (MSB) and one for the least significant bit (LSB). The circuit is biased with negative voltage (V1 = −1.2 V, V2 = −2.8 V, V3 = −1 V, V4 = −1.7 V). A simplified schematic of the proposed PAM-4 modulator can be seen in Figure 2.

Transistors Q2, Q4, Q6, and Q8 have a 12 µm emitter length, while Q1, Q3, Q5, and Q7 have an 8 µm emitter length. Q2 and Q6 are chosen to be relatively large to provide more gain for the RF output signal; Q4 and Q8 are chosen to be large for matching purposes. The LO input is fed at the base of transistors Q1 and Q5, which are both configured as emitter followers to provide a buffer between the LO input and the data input, while the data input is fed at the base of transistor Q4 for MSB, and Q8 for LSB. The voltage applied at V2 and V4 determines the amplitude change of the RF output of the corresponding ECP; thus, the MSB has a larger applied voltage at the resistor leading to the emitters of that pair. Both the LO input port and the RF output port are matched for D-band using shorted stubs.

When a bit corresponding to ‘0’ (0 V) is applied at the data input port, transistor Q4 or Q8 will be turned off, in turn also turning Q3 or Q7 off. The current will go through the Q2 and Q6 transistors, amplifying the LO input signal before delivering it to the RF output. When data ‘1’ (0.5 V) are applied at the data input port, the current will be steered away from transistor Q2 or Q6, and the LO input signal will instead be attenuated before it is delivered to the RF output port.

**Figure 3:**
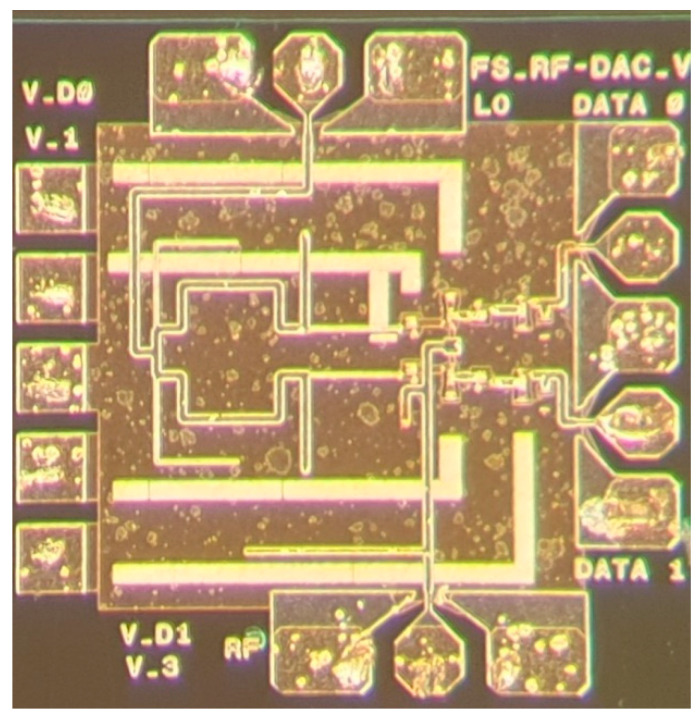
A photo of the fabricated RF-DAC. The chip size including pads is 740 µm by 740 µm.

A photo of the RF-DAC-based PAM-4 modulator can be seen in Figure 3. The LO input signal is applied on the right side and the RF output signal is delivered at the left side of the chip. Dc bias is applied on the top, while the two data input ports are located at the bottom.

The total size of the MMIC including pads is 0.55 mm^2^, and 0.29 mm^2^ without pads.

Both the PAM-4 modulator and the PD were designed and simulated using the Keysight Advanced Design System (ADS), in the same InP DHBT process.

**Figure 4:**
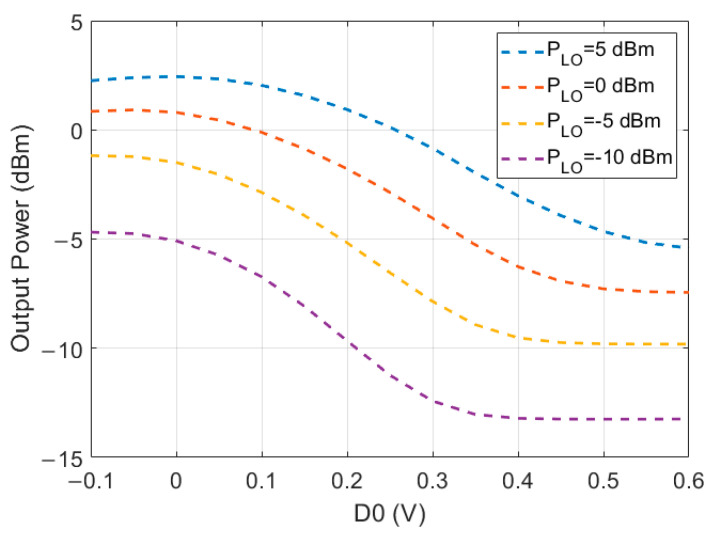
Simulated output power for different data input voltages of the MSB (D0), with different input power (LO). The LSB (D1) is set to 0 V, and the LO frequency is 130 GHz.

In Figure 4, a simulation of the output power while sweeping the data input voltages between −0.1 and 0.6 V can be seen. Four different LO power levels were used (−10 dBm, −5 dBm, 0 dBm, and 5 dBm).

### Measurement Results of the PAM-4 Modulator

**Figure 5:**
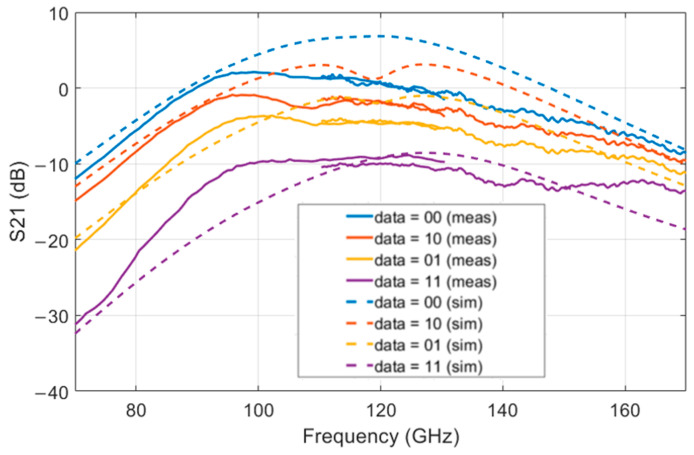
S21 for the different digital data input. LO is port 1 and RF is port 2. The measured results are shown in the solid lines and the simulated results in the dashed lines. The LO input power is −6 dBm.

The PAM-4 modulator was measured in frequency domain, on-wafer, using a MPI TS200 probe station. The output S21 measurement was captured with an Anritsu VectorStar ME7838A vector network analyzer (VNA). A frequency sweep from 70 GHz to 130 GHz and 130 GHz to 170 GHz can be seen in Figure 5. The reason for the two separate measurements is that different VDI VNA extenders were used. The four different traces represent the four different input data states of the PAM-4 modulator. The highest level corresponds to data input ‘00’, while the lowest level corresponds to data input ‘11’.

The −3 dB bandwidth is between 87 GHz and 130 GHz, resulting in a 43 GHz wideband −3 dB bandwidth. The RF-DAC is still usable outside of this band, but it provides less output power. The difference in power between the highest power level ‘00’ and the lowest power level ‘11’ is approximately 10 dB for frequencies between 90 and 145 GHz.

**Figure 6:**
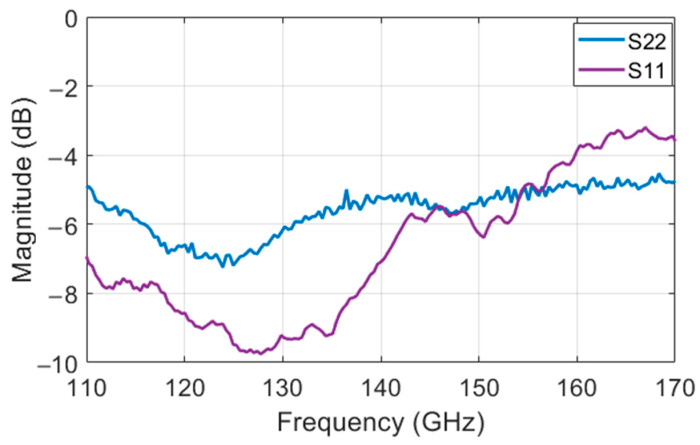
S11 and S22 for the modulator for different frequencies.

Input and output matching of the modulator can be seen in Figure 6.

**Figure 7:**
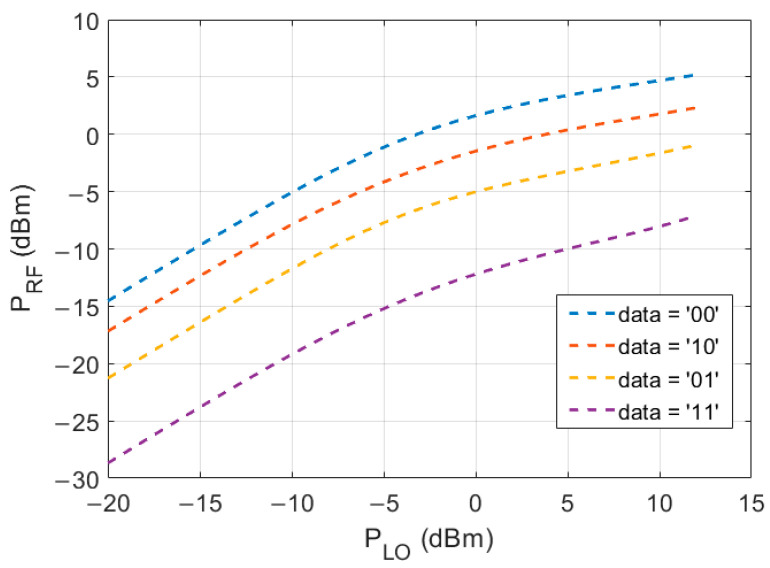
ADS simulation result of the RF output power level depending on the LO input power. The LO frequency is 130 GHz.

A Keysight Advanced Design System (ADS) was used to simulate and estimate the RF output power for different LO input power levels. This relationship is plotted in Figure 7. The four different traces correspond to the four different PAM-4 data input power levels. The RF output power saturates around 5 dBm output power, according to this simulation. An LO input frequency of 130 GHz was used.

The DC power consumption of the RF-DAC was measured to be 112 mW.

## 3. Design of the Power Detector

**Figure 8:**
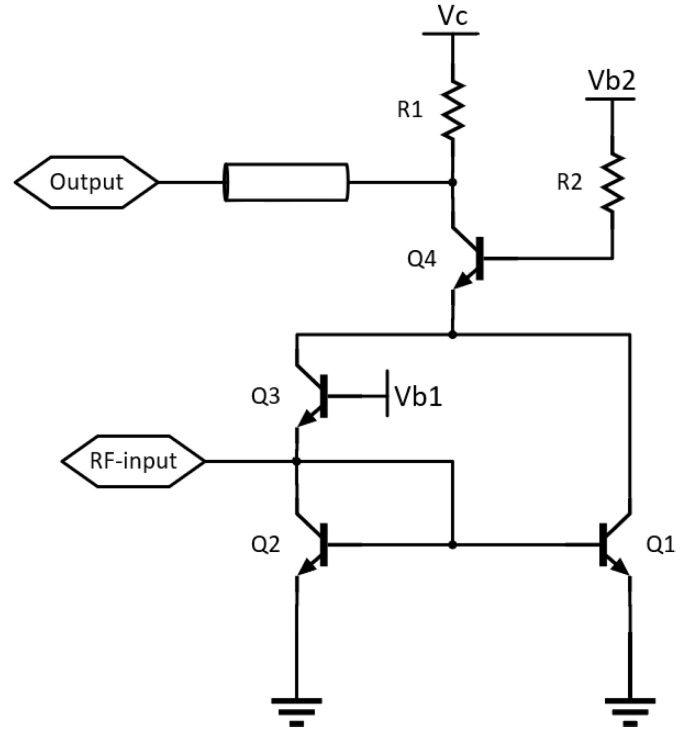
A schematic of the power detector.

As shown in Figure 8, a common-emitter configured transistor, Q1, and a common-base configured transistor, Q3, are used as nonlinear components for power detectors. Their collector currents are added and applied at the stacked transistor Q4, to amplify the output signal. The output of the power detector is taken at the collector of Q4. The collector and base of Q2 are connected to form a diode, which provides a DC path for Q3, and DC-bias for Q1.

The collector current of a transistor is given by

*I*_c_ = *a*_0_ + *a*_1_*V_be_* + *a*_2_*V*^2^ + … (1)

where *a*_0_ is a function of amplitude of *V_be_* in an ideal case, *V_be_* for common-emitter configured transistor is equal to *−V_be_* for commonbase configured transistor.

The single-ended input signal is applied at the base of Q1 and the emitter of Q3 simultaneously. Due to the transistor’s nonlinearity, the outputs of Q1 and Q3 contain DC component and harmonics. The odd-order harmonics at Q1 and Q3‘s outputs are 180 degrees out of phase, since the transistor Q1 is common-emitter configured and Q3 is common-base configured. Thus, connecting Q1 and Q3′s outputs, the odd-order harmonics will be suppressed. This is a desired feature for a power detector, since all harmonics create ripple at the output waveform; among them, the 1st harmonic makes the most contribution [6].

The emitter length of transistor Q4 is 15 µm, and for Q1, Q2, and Q3, the emitter length is 3 µm. The desired function of Q4 is amplification of the data signal, which is why a relatively large device is selected to get sufficient gain. Due to parasitic action, the device will have a lower gain for the RF-signal, which will help to suppress undesired fundamental and higher order harmonics. Three different bias voltage supplies are used (Vc = 4.5 V, Vb2 = 2.2 V, Vb1 = 1.5 V).

**Figure 9:**
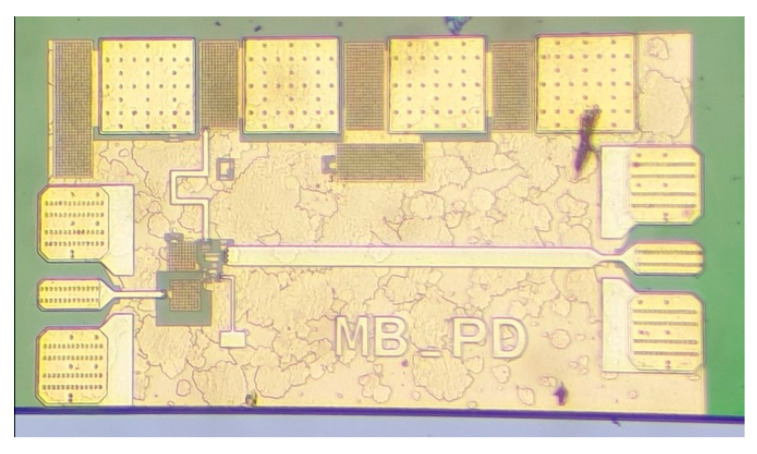
A photo of the fabricated power detector. The chip size including pads is 700 µm by 400 µm.

A photo of the fabricated PD can be seen in Figure 9. The RF input signal port is located on the left side of the MMIC, while the output port is located on the right side. The DC bias is supplied at the top. The total size of the MMIC including pads is 0.28 mm^2^, and 0.11 mm^2^ without pads.

The PD was measured on wafer. The input sinusoidal signal was swept over frequency and input power using a Keysight N5261A PNA-X microwave network analyzer.

**Figure 10:**
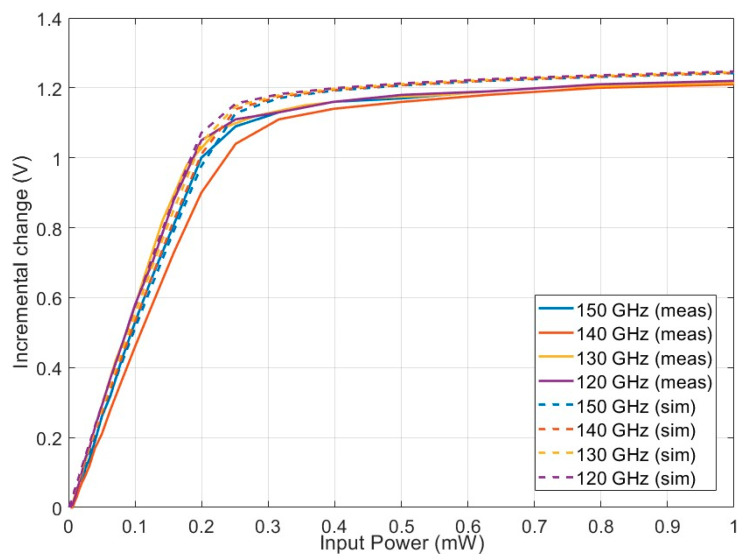
Incremental output voltage change for different input powers applied. Each trace corre sponds to a different frequency (120 GHz, 130 GHz, 140 GHz, and 150 GHz).

The output of the PD (DC voltage) was measured by a multimeter. The output voltage corresponding to different input power is plotted in Figure 10 for 120 GHz, 130 GHz, 140 GHz, and 150 GHz. As the input power increased from 0 to 1 mW, the output voltage changed 1.1 V, which agreed with the simulation.

The DC consumption for the PD was measured to be 11 mW.

**Figure 11:**
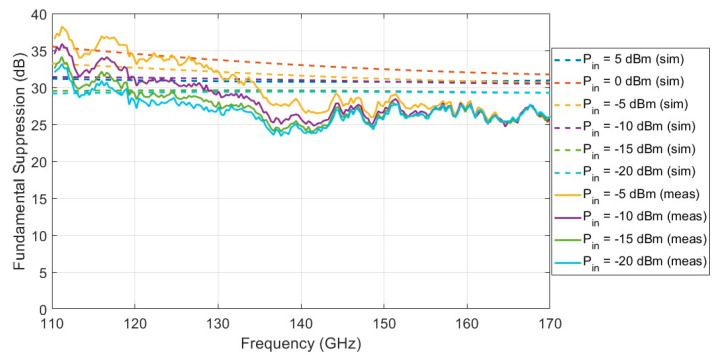
Simulated and measured suppression of the fundamental at the output of the PD. Six different values for the input power were used, from −20 dBm to 5 dBm during the simulation, and four different values for the input power were used, from −20 dBm to −5 dBm during the measurement.

The suppression of the fundamental frequency at the output of the PD was measured and simulated using ADS. The measured suppression is more than 24 dB for the entire D-Band. The results can be seen in Figure 11.

**Figure 12:**
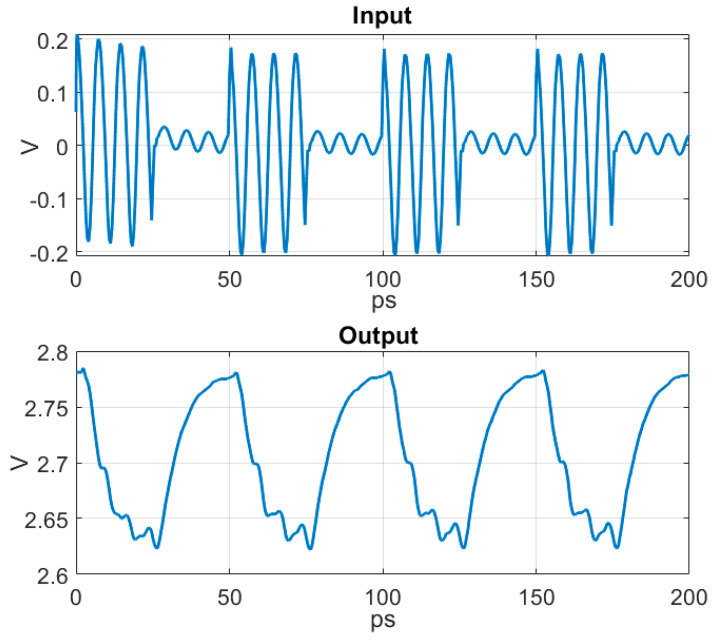
Time domain simulated input and corresponding output from the PD. The carrier frequency is 140 GHz.

A time domain simulation of the input signal and corresponding output signal can be seen in Figure 12. The carrier frequency was 140 GHz.

## 4. Measurement Results of the PMF Link

**Figure 13:**
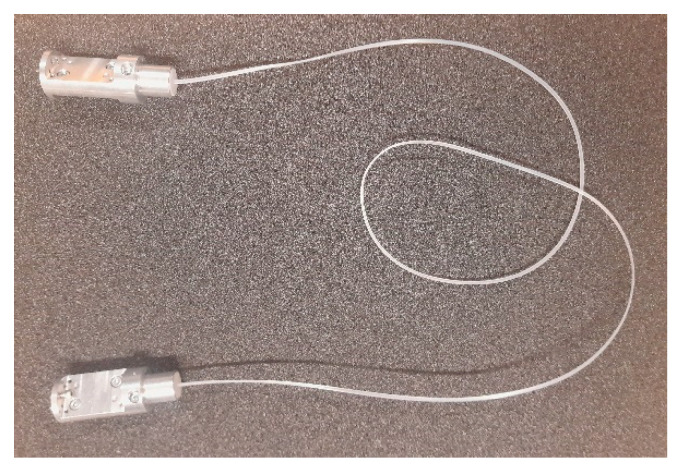
The polymer microwave fiber that was used in the test setup.

**Figure 14:**
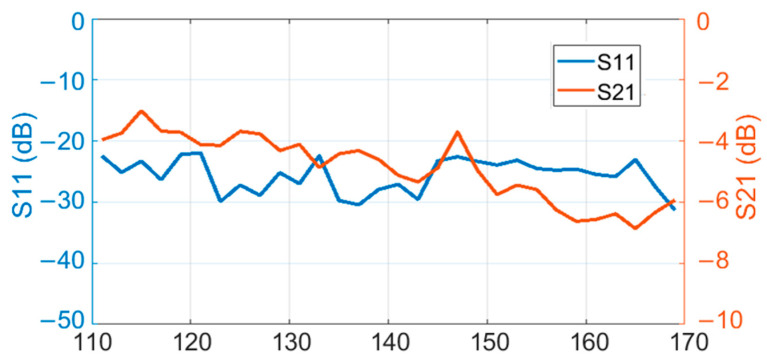
Measured reflection loss and transmission loss of the fiber that was used during the measurements.

**Figure 15:**
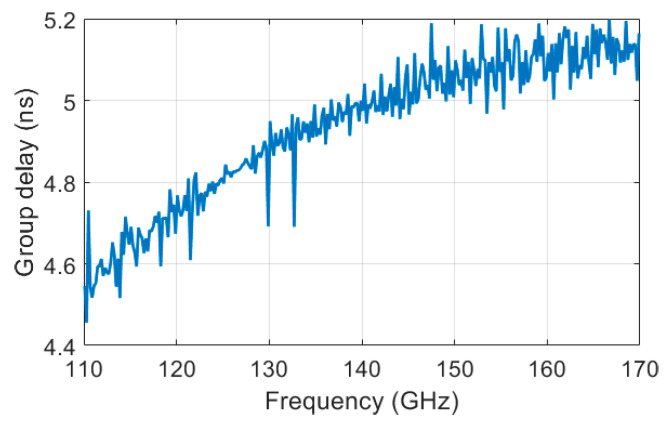
Measured group delay of the fiber that was used during the measurements.

The polymer microwave fiber that was used during these measurements was provided by Lehrstuhl für Hochfrequenztechnik (LHFT), Germany. A photo of the D-band dielectric waveguide can be seen in Figure 13. The length of the fiber is 1 m and it has a rectangular solid cross section (1 mm by 2 mm). Measured reflection loss and transmission loss of the fiber, including transitions from the waveguide to fiber, are shown in Figure 14. The group delay can be seen in Figure 15. Both measurements include the fiber-to-waveguide transitions.

**Figure 16:**
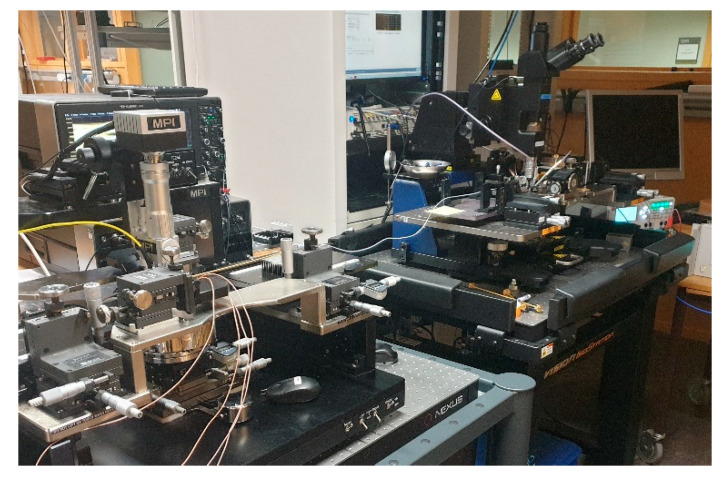
The setup that was used in the PMF link measurements. The fiber can be seen connecting one circuit on one probe station to the other one on the other probe station.

The entire link was tested in the time domain, transferring data in real time. The output from the PD was then connected using a coaxial cable to a Lecroy LabMaster 10–100 Zi real-time oscilloscope, where the signal could be analyzed. A photo of the fiber connecting the circuits on each probe station can be seen in Figure 16.

The LO input signal for the RF-DAC was provided by a VDI vector network analyzer (VNA) through an extender WR 6.5. The LO input frequency was set to 130.6 GHz, which together with the probe loss results in an input power of approximately −1.5 dBm delivered at the input of the RF-DAC. The data input was provided by a Keysight M8195A arbitrary waveform generator (AWG).

**Figure 17:**
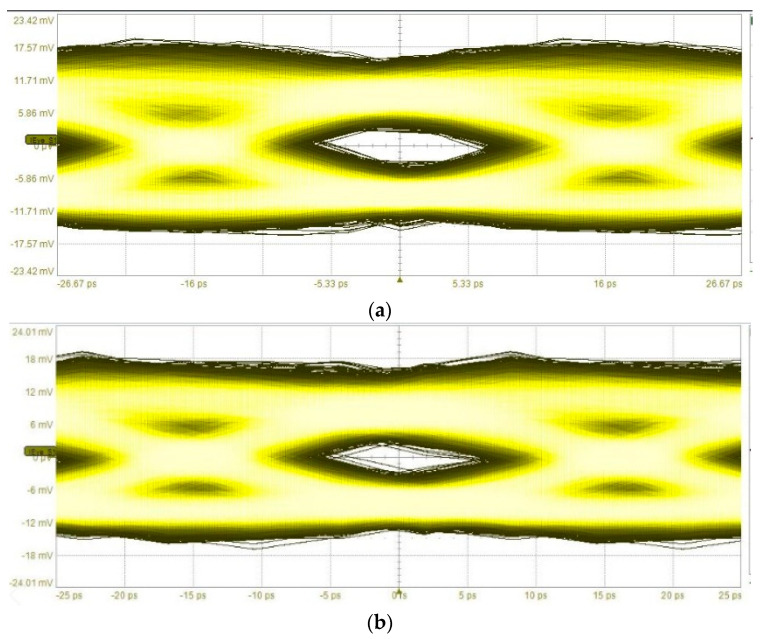
Eye diagrams from the demodulated PAM-2 signal at 130.6 GHz. (**a**) 30 Gbps signal. BER < ×10^−12^. (**b**) 32 Gbps signal. BER = 2.6 × 10^−10^.

First, the PMF link was tested using only one data input (MSB) to create a PAM-2 modulated signal. Bit rates up to 32 Gbps were measured and the data input that was used was a PRBS-9 stream. The eye diagram of the output signal from the PD can be seen in Figure 17a for a 30 Gbps signal and in Figure 17b for a 32 Gbps signal. For a 32 Gbps signal, the bit error rate (BER) was 2.6 × 10^−10^.

**Figure 18:**
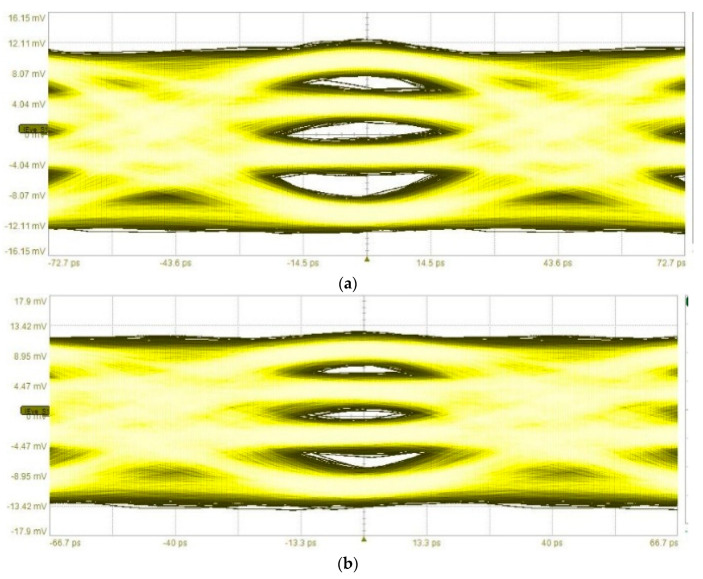

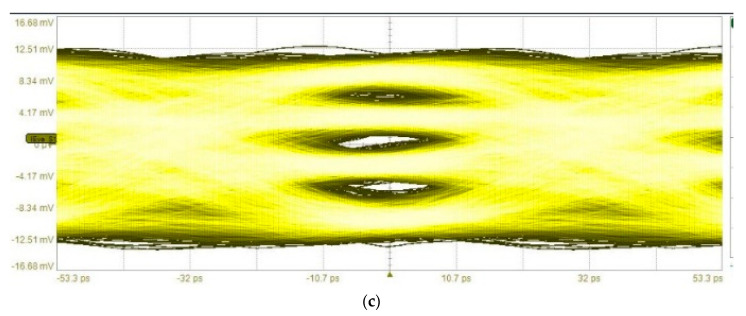
Eye diagrams from the demodulated PAM-4 signal. (**a**) 11 GBaud signal. BER < 10^−12^. (**b**) 12 GBaud signal. BER = 2.8 × 10^−12^. (**c**) 15 GBaud signal. BER = 4.3 × 10^−10^.

For the PAM-4 PMF link measurements, a PRBS-9 stream was used at the MSB data port and a PRBS-10 stream was used at the LSB data port. Data rates up to 15 GBaud were tested, corresponding to 30 Gbps. For 30 Gbps PAM-4 transmission, the BER was 4.3 × 10^−10^. Eye diagrams of the measurements can be seen in Figure 18.

The total DC power consumption for the PMF link was measured to be 123 mW, which results in an energy efficiency of 4.1 pJ/bit for a 30 Gbps data transmission.

**Figure 19:**
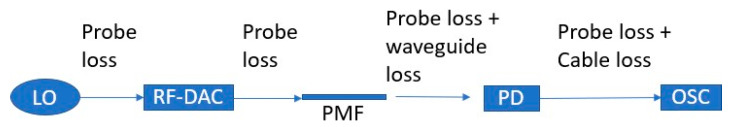
Overview of the link.

An overview of the link is displayed in Figure 19, and an estimation of the link budget can be seen in Table 1.

The noise floor of the oscilloscope is approximately −73 dBm.

## 5. Discussion and Conclusions

The communication link presented in this paper uses a cost-efficient plastic fiber to transfer ultra-high data rates. A demonstration was performed over a one-meter fiber. In the measurements, the input power was close to the detection limit for the PD, which is why the system at the time was limited by power. Therefore, for longer fibers or a higher performance, a low noise amplifier (LNA) can be placed before the power detector. Adding an LNA will contribute with more noise, decreasing the signal-to-noise ratio (SNR), which eventually becomes a limitation. Since the signal has a wide bandwidth, due to low modulation complexity, dispersion of the fiber cannot be neglected for longer fibers. Comparing the difference in group delay over the signal’s bandwidth with the baud rate will give an indication when symbol interference becomes problematic.

In Table 2, the proposed PMF link is compared to previously published PMF links. It can be seen that a highly energy-efficient transmission (4.0 pJ/bit) over PMF was achieved in [3], as well as a high data rate (17.7 Gbps). In this work, 30 Gbps (BER < 10^−12^) was demonstrated, in real-time over a one-meter long PMF, which is the highest data rate. Moreover, the transmitter and receiver have the smallest combined chip area of 0.83 mm^2^, and an energy efficiency of 4.1 pJ/bit, which is competitive considering that it does not include an LO. The proposed link can be used for ultra-high data rate, short-range communication (device-to-device, chip-to-chip etc.), for example within a car.

## Figures and Tables

**Table 1:** Link budget: carrier frequency at 131 GHz.

Stage	Change	Power
LO	-	0 dBm
Probe loss	−1.5	−1.5 dBm
RF-DAC	−3	−4.5 dBm
Probe loss	−1.5	−6 dBm
PMF	−5	−11 dBm
Waveguide bends/transitions	−2	−13 dBm
Probe loss	−1.5	−14.5 dBm
PD	−1.5	−16 dBm
Probe loss	−0.5	−16.5 dBm
Cable loss	−1	−17.5 dBm

**Table 2:** Comparison with other PMF links, with BER < 10^−12^.

Modulation	Frequency (GHz)	Data Rate (Gbps)	Fiber Length (m)	Energy Eff. (pJ/bit) (* = no LO) Included	Total Chip Area (mm^2^)	Reference
CP-FSK	120	17.7	1.0	4.0 *	N/A	[3]
CP-FSK	140	12	1.0	19.2	2.31	[7]
ASK	60	6	2.0	4.7	N/A	[8]
ASK	135	27	1.0	4.8	1.94	[9]
PAM-2	131	30	1.0	4.1 *	0.83	this work
PAM-4	131	22	1.0	5.6 *	0.83	this work

## Data Availability

Not applicable.

## Abbreviations

The following abbreviations are used in this manuscript:

BER	Bit error rate
PAM	Pulse amplitude modulation
PMF	Polymer microwave fiber
PD	Power detector
HD-PE	High-density polyethylene
AWG	Arbitrary waveform generator
RF-DAC	Radio frequency digital to analog converter
LSB	Least significant bit
MSB	Most significant bit
